# UCH-L1 regulates eye differentiation-related genes and modulates EGFR signalling in *Drosophila melanogaster*

**DOI:** 10.1080/19336934.2025.2580003

**Published:** 2025-11-13

**Authors:** Nguyen Anh Tuan, Tran Linh Thuoc, Dang Thi Phuong Thao

**Affiliations:** a Department of Molecular and Environmental Biotechnology, Faculty of Biology and Biotechnology, University of Science, Ho Chi Minh City, Vietnam; b Vietnam National University, Ho Chi Minh City, Vietnam; c Laboratory of Molecular Biotechnology, University of Science, Ho Chi Minh City, Vietnam

**Keywords:** UCH-L1, eye develoment, photoreceptor cells, differentiation, EGFR signaling pathway

## Abstract

UCH-L1 (Ubiquitin Carboxyl-terminal Hydrolase – L1) is a protein that plays a critical role in the ubiquitin-proteasome system. Previous studies have demonstrated a link between UCH-L1 and various diseases, including neurodegenerative disorders, diabetes, and cancer. However, the role of UCH-L1 in development remains unclear. To investigate the functions of UCH-L1 in a living organism, taking advantage of the *Drosophila* model, and to explore the correlation between Drosophila UCH (dUCH) and human UCH-L1, we established a GAL4/UAS-targeted expression system to examine the effect of dUCH on *Drosophila* eye development. We found that knockdown of dUCH resulted in a rough eye phenotype associated with the MAPK pathway. In this study, for the first time, we revealed that loss of dUCH function leads to a reduction in EGFR protein levels. Additionally, dUCH knockdown downregulated Spitz (spi), a ligand of EGFR, as well as *Draf*, a key component of the MAPK pathway. Furthermore, under dUCH knockdown conditions, several genes known to play critical roles in eye cell differentiation were affected, including the downregulation of sens, salm, lz, barth1/2, and salm, which are essential for the differentiation of R2/5, R3/4, and R1/6 photoreceptor cells. Interestingly, dUCH was found to be involved not only in the MAPK pathway but also in the regulation of *pros, lz, barth1/2, and sev* gene expression, suggesting its role in R7 photoreceptor differentiation. Taken together, these findings highlight the important role of dUCH in regulating genes associated with eye cell differentiation and its involvement in EGFR signalling in *Drosophila melanogaster*.

## Introduction

Ubiquitin C-terminal Hydrolase L1 (UCH-L1, also known as PGP 9.5) is a 223 amino acid, brain-specific human protein that accounts for approximately 1–2% total human brain proteins. Its expression is about 50-fold higher in the brain compared to other tissues such as kidneys, prostate, large intestine, testis [1–3]. UCH-L1 is a protease classified within the deubiquitinating (DUB) enzyme superfamily, which is a component of the ubiquitin-proteasome system (UPS). It functions by cleaving the bond between ubiquitin and its substrate, thereby playing a key role in the protein ubiquitination and turnover within the cell [4,5].

Although the specific mechanism of UCH-L1 remain unclear, previous studies have shown that UCH-L1 plays essential roles in various cellular processes, particularly in neuronal cells. Dysfunctional mutations of *uch-l1* have been identified in several neurodegenerative diseases [6–15]. Additionally, UCH-L1 has been implicated in various types of cancer, including non-small cell lung cancer [16,17], colorectal cancer [18,19], breast cancer [20], prostate and ovarian cancer [21,22]. However, the precise roles of UCH-L1 in cellular function and development are still not fully understood.

*Drosophila* eye development offers several advantages for studying the cellular mechanism of specific molecules. The *Drosophila* eye is a compound structure composed of approximately 700–800 ommatidia. Each ommatidium consists of eight photoreceptor cells, four cone cells, twelve pigment cells, and other supporting cell types. The photoreceptor cells, named R1 to R8, are categorized into outer and inner photoreceptors based on their location, morphology, and function [23]. Cells R1 to R6 are classified as outer photoreceptor, characterized by their long rhabdomere and broad-spectrum light sensitivity, making them essential for motion detection and vision in low-light conditions [23]. In contrast, R7 and R8 are inner photoreceptor cells, possessing short, stacked rhabdomere, and are primarily involved in colour discrimination [23].

Starting from a monolayer of epidermal cells, these cells undergo numerous proliferation and patterning steps that are precisely regulated by both planar and peripheral signals. Initially, differentiation begins at the posterior border of the eye imaginal disc, also known as the morphogenetic furrow, and progresses anteriorly. R8 cells differentiate first, followed by the specification of R3/R4 cells; this group of five cells is known as the precluster. Subsequently, the remaining cells undergo a second mitotic wave of proliferation before continuing to differentiate into R1/R6, R7, cone cells, pigment cells, and other cell types [24–27]. The development of photoreceptor cells is tightly regulated by the expression of R cell-specific transcription factors, including Atonal in R8 cells [28–30]; Rough in R2/R5 cells [31]; Seven-up and Spalt-major in R3/R4 [32–34]; Seven-up and BarH1/2 in R1/6 [32,33,35,36]; and Prospero, Sevenless in R7 cells [37,38]. These processes are coordinated by several signalling pathways, including the Spitz/EGFR/MAPK and Delta/Notch [24–26,39–41].

The EGFR pathway is crucial for the recruitment of ommatidial cells other than R8, primarily through the action of EGFR ligands such as Spitz (Spi) and downstream signalling cascades including Ras – Raf – MAPK [42]. Both under-activation and over-activation of the EGFR pathway adversely effect the recruitment of photoreceptors, cone cells, and pigment cells [43].

The involvement of UPS in *Drosophila* eye development has been previously reviewed [44]. Several E3-ubiquitin ligases, such as SORDD1/2, have been showed to play important roles in maintaining rhodopsin homoeostasis and integrity of photoreceptor cells [45]. Cullin-4 has also been implicated in *Drosophila* eye differentiation by regulating the Wingless, JNK, and Homothorax signalling pathways [46,47]. The role of another E3-ubiquitin ligase, SINA, in R7 photoreceptor differentiation is also established [48,49]. Among the deubiquitinating enzymes (DUBs) involved in the regulation of the *Drosophila* eye, UBP64 has been suggested to counteract the effects of SINA, while USP5 has been shown to regulate both the Notch and receptor tyrosine kinase (RTK) signalling pathways [50].

Our previous results demonstrated that dUCH overexpression affects *Drosophila* eye development through the MAPK signalling pathway and apoptosis [51]. In the present study, we explore the effects of dUCH loss-of-function and observe that depletion of dUCH leads to a marked decrease in EGFR protein levels. This reduction subsequently downregulates Draf activity within the MAPK pathway. As a downstream effect, several genes critical for photoreceptor cell differentiation are also misregulated in response to dUCH knockdown

## Methods

### Fly stocks

Fly stocks were maintained at 25°C on standard food containing 0.7% agar, 5% glucose, and 7% dry yeast. The dUCH knockdown (*UAS-dUCH IR*) fly strain (V26468) was obtained from the Vienna Drosophila RNAi Center (VDRC). The transgenic fly line carrying GMR-GAL4 on the X chromosome (strain number 16) was described previously [52].

### Immunostaining

For immunohistochemistry, larval eye imaginal discs were dissected and fixed in 4% paraformaldehyde in PBS for 20 minutes at 25°C. After three washes with phosphate-buffered saline (PBS), the samples were blocked in 10% normal goat serum diluted in 0.3% Triton X-100 in PBS for 30 minutes at 25°C, and then incubated with primary antibodies diluted in 0.15% Triton X-100 in PBS for 16 hours at 4°C.

The following primary antibodies were used independently: anti-dUCH (1:500) and anti-dEGFR (1:500, Sigma, E2906). After extensive washing with PBS containing 0.3% Triton X-100, samples were incubated with secondary antibodies conjugated to either Alexa Fluor 594 or Alexa Fluor 488 (1:400, Invitrogen) for 2 hours at 25°C. Following additional washes with PBS containing 0.3% Triton X-100 and PBS alone, the samples were mounted using Vectashield Mounting Medium (Vector Laboratories).

Fluorescent images were acquired using an Olympus BX-50 fluorescence microscope equipped with a cooled CCD camera (ORCA-ER, Hamamatsu Photonics).

### Scanning electron microscopy

The eye morphology of adult files was examined using electron microscopy. Adult flies were anesthetized, mounted on sample stages, and imaged using a VE-7800 scanning electron microscope (Keyence Inc.) in low vacuum mode. Rough eye area was quantified using Image J software.

### Quantitative pcr

For qPCR analysis, 240 eye imaginal discs of the third late larvae (~4.5 days after laying eggs) were dissected per fly line. Total RNA was extracted from the eye discs using TRIsure™ reagent (Meridian) following the phenol – chloroform extraction method. The extracted RNA was then reverse transcribed into cDNA using the PrimeScript™ RT Reagent Kit (Takara). The concentrations of RNA and cDNA were measured using a NanoDrop 1000 spectrophotometer (Thermo Fisher Scientific). The following primers were used for qPCR: dRP49 reference gene F-R (5’- AGATCGTGAAGAAGCGCACC; 5’- CGATCCGTAACCGATGTTGG), EGFRF_R (5’- TGGCGATCGTTAAGTCATCCCTGT; 5’- TGCACTGATCCGAGCAAATGGTTC), spi F-R (5’- TTGTTGTCATATACACACATCACCC, 5’- TGCTAGGGCGTCGTCAAATC), star F-R (5’- CTGAATCCCTCGCCCTATCG, 5’- TGGCGAATCTCTGTCGTGTC), rho F-R (5’- GAACTAATCGCCTCTCGCTATG, 5’- CAGTTTGCTGATGCTTCGATTC), draf F-R (5’- CCACGAGCACCTTGAAACAC, 5’- CCGCCAGAATATTCCAGTTTTCC), ato F-R (5’- CGTCGGATGCAGAACCTCAA, 5’- TCCCCGAGAGCGGATATGTA), boss F-R (5’- CCTTATTCGGGGACGAGTGG, 5’- AAGCCAGCGAAGTGAGTGAA), ro F-R (5’- CTTTTCCGCATTTCTGGCTCG, 5’- TGGAGATGTACTCGTTCCGATG), sens F-R (5’- ATACATACCGGCGAGAAGCC, 5’- ACTGATCGCAAAGGTGGCAA), svp F-R (5’- ATGAGACGCGAAGCTGTTCA, 5’- TACGAGTGCCCGTTAAAGCC), salm F-R (5’- TTCTGTGTGCGATGCGTAGT, 5’- CTACCGATGTCTTTGTCGGCG), barh1 F-R (5’- CGGCAGAAGTACCTTAGCGT, 5’- CATTTGGTTCTGCGGTTTTGGT), barh 2 F-R (5’- GGTACCAGAATCGCAGAACCA, 5’- TATGGCCAGGCGCTCAAATAG), pros F-R (5’- ACGACAAGCTGTCACCGAAG, 5’- AATTCTGGGGTACCTCAATGTG), sev F-R (5’- GAGTTGCTTTTCAGTTGGGGC, 5’- AACTCTCACCCACATTTCGCT), lz F-R (5’- ATGATCTGCGTTTCGTGGGA, 5’- ACGGTCACCTTGATGGCTTT)

Quantitative PCR (qPCR) was performed using the LightCycler® 96 System (Roche) with the SensiFAST™ HRM Kit (Bioline). Only amplification products showing a unique high-resolution melt (HRM) peak were included in the analysis to ensure specificity. Reactions with Cq values ≥38 cycles were considered as failed amplifications and excluded from further analysis. Each qPCR reaction was conducted in triplicate. The final gene expression levels were calculated using the ΔΔCq method [53], with dRP49 serving as the internal reference gene. Data analysis and relative quantification were performed using LightCycler® 96 software (Roche) and GraphPad Prism for statistical processing and visualization.

### Data analysis

Statistical analyses and graph generation were performed using GraphPad Prism version 8.0.2 (GraphPad Software, San Diego, CA, USA). Unpaired two-tailed *t*-tests were used to compare differences between groups. A p-value less than 0.05 was considered statistically significant. Data are presented as the mean ± standard deviation (SD) from independent biological replicates in each group.

## Results

### The loss function of dUCH disrupted normal eye development in Drosophila melanogaster

In an effort to utilize *Drosophila melanogaster* as a model organism to investigate the potential functions of UCH-L1 in vivo, we employed the GAL4/UAS targeted expression system to examine the effects of dUCH loss-of-function on *Drosophila* development [54]. Since *Drosophila* imaginal discs serve as a powerful model for studying various aspects of development, we utilized the GMR-GAL4 driver, which enables dUCH knockdown from the morphogenetic furrow to the posterior region of the eye imaginal disc – where cell proliferation, differentiation, and programmed cell death occur to form the mature ommatidia of the adult fly eye [54], This system was employed to investigate the effect of dUCH knockdown in *Drosophila melanogaster*.

**Figure 1.**
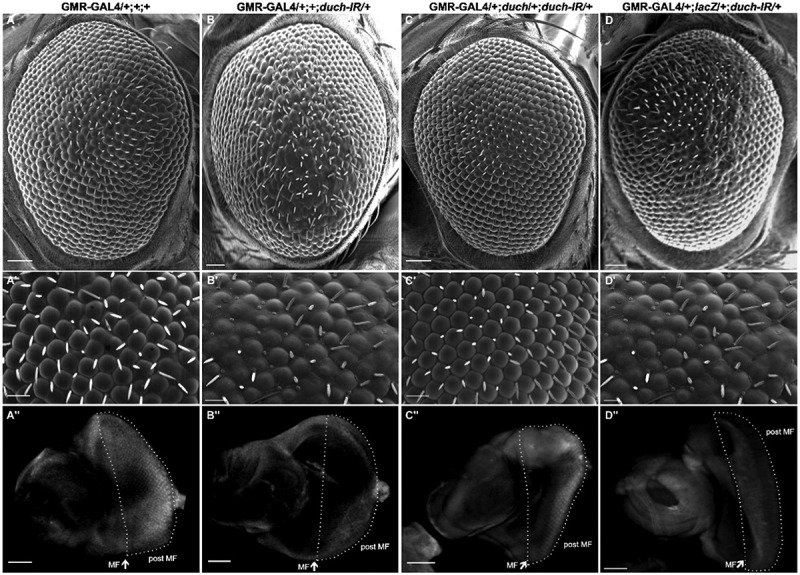
Knockdown d*uch* caused rough eye phenotype in *Drosophila*. A-C’: the eye phenotype captured by sem. A’’-C’’: the dUCH expression level in eye imaginal discs were observed by immunostaining with anti-dUCH antibody. A, A’, A’’. The control line: flies carry GMR-Gal4 driver; B, B’, B’’. The knockdown d*uch* line: flies carry GMR-Gal4 and UAS-duch IR; C, C’, C’’. The rescued knockdown line: flies carry GMR-Gal4, UAS-duchIR and Uas-duch. D, D’, D’’. The other control line: flies carry GMR-Gal4, UAS-duchIR and Uas-lacZ. Scale bar: 50µm.

A previous study demonstrated that overexpression of dUCH resulted in a rough eye phenotype in Drosophila [51]. Consistent with this, our results revealed that loss-of-function of dUCH also led to a rough eye phenotype in all adult Drosophila melanogaster examined (Figure 1(A,A′,B,B′)). As expected, overexpression of dUCH successfully rescued the rough eye phenotype induced by dUCH knockdown (Figure 1(C,C′)). All of the dUCH RNAi flies had a normal eye phenotype when dUCH was restored by GMR-Gal4/UAS-dUCH, whereas overexpression of lacZ in the control fly line GMR-Gal4/+; lacZ/+; dUCH-IR/+ did not produce any rescuing effect on dUCH loss (Figure 1(D,D′)). The results shown in Figure 1(D,D′) further support that the rescued phenotype observed in Figure 1(C,C′) was due to restoration of dUCH expression levels in the knockdown background, rather than being caused by GAL4 titration, including potential reduction of GAL4 activity due to the presence UAS constructs.

Interestingly, when dUCH knockdown is driven by Rh1-Gal4, which is known to be specifically expressed in outer photoreceptors R1–R6, the result is a loss of eye pigmentation without any accompanying rough-eye phenotype. The similar results of pigmentation loss was also observed when Rh3-Gal4, Rh3-Gal4 and Rh6-Gal4 drivers were utilized (supplementary Figure S1).

Taken together, these results strongly indicate that reduced expression of dUCH disrupts normal eye development in Drosophila melanogaster (Figure 1(A-C)).

### Enhancement of Draf rescued rough eye phenotype induced by loss function of dUCH

The RTK/Ras/MAPK signalling pathway is essential for the specification of various ommatidial cell types in the *Drosophila* eye. Within this cascade, Draf – the *Drosophila* homolog of Raf – functions as a critical kinase that transmits signals downstream of Ras activation. Upon recruitment to the membrane by Ras, Draf phosphorylates and activates MAPK (Rolled), which in turn regulates the expression of transcription factors involved in photoreceptor differentiation. Mutations or downregulation of Draf disrupt this signalling flow, leading to improper ommatidial patterning and failure in the differentiation of specific photoreceptor cells, particularly R1-R7. These findings highlight the pivotal role of Draf as a central effector in mediating EGFR-dependent developmental signals during eye morphogenesis [55,56].

**Figure 2.**
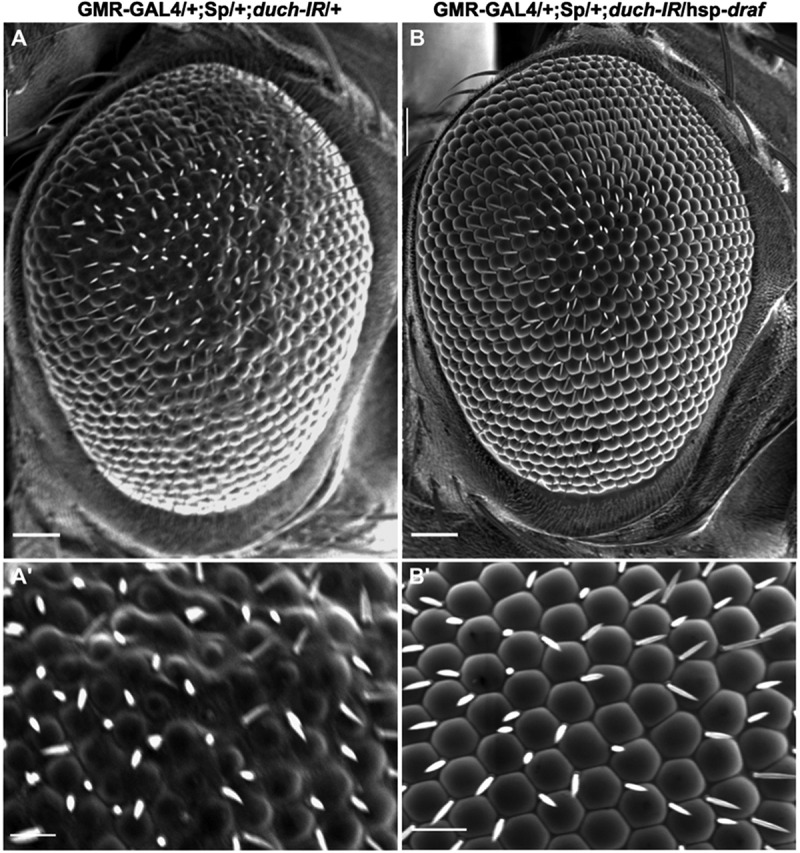
Overexpression Draf rescued rough eye phenotype induced by knockdown d*uch*. A, A’: the knockdown d*uch* line: flies carry GMR-Gal4 and UAS-duch ir; B, B’: the Draf enhancement line: flies carry GMR-Gal4, UAS-*duch*IR, and hsp-*draf.*

To investigate the involvement of dUCH in the MAPK pathway, we enhanced pathway activity in dUCH knockdown flies by overexpressing Draf. The results demonstrated that dUCH influences the MAPK pathway, as the rough eye phenotype induced by dUCH knockdown was rescued by Draf overexpression (Figure 2).

### Effect of dUCH knocked-down in expression of EGFR and EGFR components

**Figure 3.**
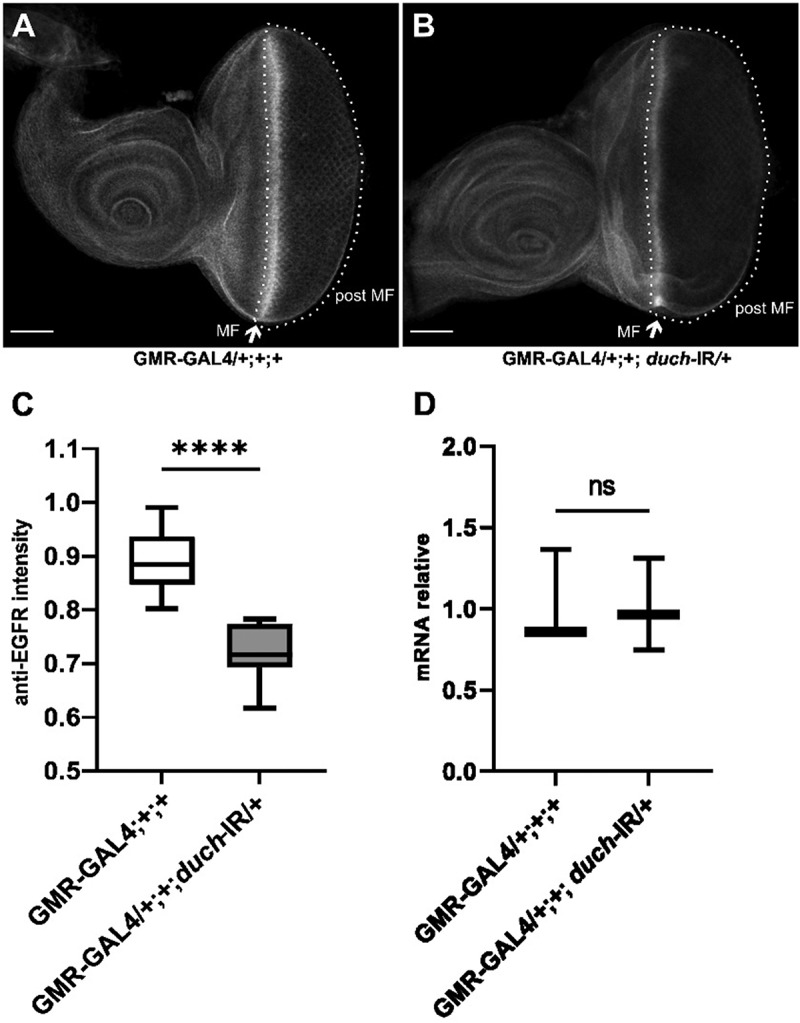
EGFR expression in knockdown d*uch* third larval eye imaginal disc. A: the control line: flies carry GMR-Gal4 driver; B. The knockdown d*uch* line: flies carry GMR-Gal4 and UAS-duch IR. C: comparing of EGFR protein level between control (*n*=12) and knockdown d*uch* line (*n*=17), *t*-test (*p*<0.0001). D. comparing of *egfr* mRNA level between control and knockdown duch line, *t*-test (*p*=0.94).

To further investigate this interaction, we performed immunostaining using an anti-EGFR antibody and observed a reduction in EGFR protein levels in *duch* knockdown flies compared to control flies (Figure 3(A–C)). However, no significant difference was detected in *egfr* transcript levels (Figure 3(D)).

**Figure 4.**
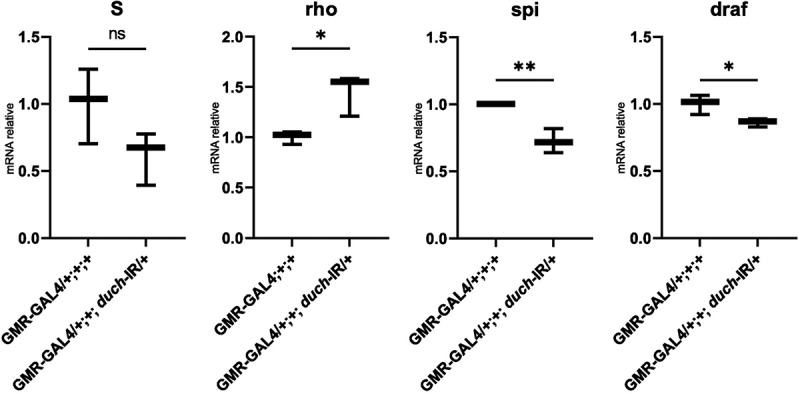
Expression level of EGFR pathway components. GMR-Gal4/+;+;+: control fly. GMR-Gal4/+;+;duch=IR/+: knockdown duch fly. *t-*test analysis with S (*p*=0.12), rho(*p*=0.02), spi (*p*=0.006), d*raf* (*p*=0.04).

To further investigate the involvement of dUCH in the EGFR pathway, we analysed the expression of several upstream and downstream components of this signalling cascade. qPCR results revealed a significant decrease in the expression of *Spitz*, the ligand of EGFR, in the *duch* knockdown line compared to the control. A slight, non-significant reduction in *Star* expression was also observed. Interestingly, *Rhomboid* expression was upregulated under low dUCH conditions. Consistent with the reduced expression of *Spitz* and EGFR, the downstream EGFR pathway component *Draf* was also downregulated (Figure 4).

### Involvement of dUCH knocked-down on eye cell differentiation-related gene expression

The EGFR signalling pathway plays a critical role in *Drosophila* eye development by regulating eye cell differentiation [57,58]. Based on our previous findings, which showed that dUCH influences the expression of EGFR and its pathway components, we further examined the effect of *duch* knockdown on the transcription of key factors involved in eye cell differentiation

A reduced level of dUCH led to significant changes in the transcription of key factors involved in photoreceptor cell development. Notably, *rough (ro)*—a crucial transcription factor required for normal ommatidial development in R2/R5—was upregulated. In contrast, *senseless (sens)*, which is directly bound and repressed by Rough [31], showed decreased expression in the *duch* knockdown context. While the expression of *seven-up (svp)*—essential for preventing R3/4/1/6 precursors from differentiating into R7 or R8 [32,33], remained unchanged, the expression of *spalt major (salm)*, a key factor for establishing planar polarity in R3/4 [34], was reduced upon loss of dUCH function.

**Figure 5.**
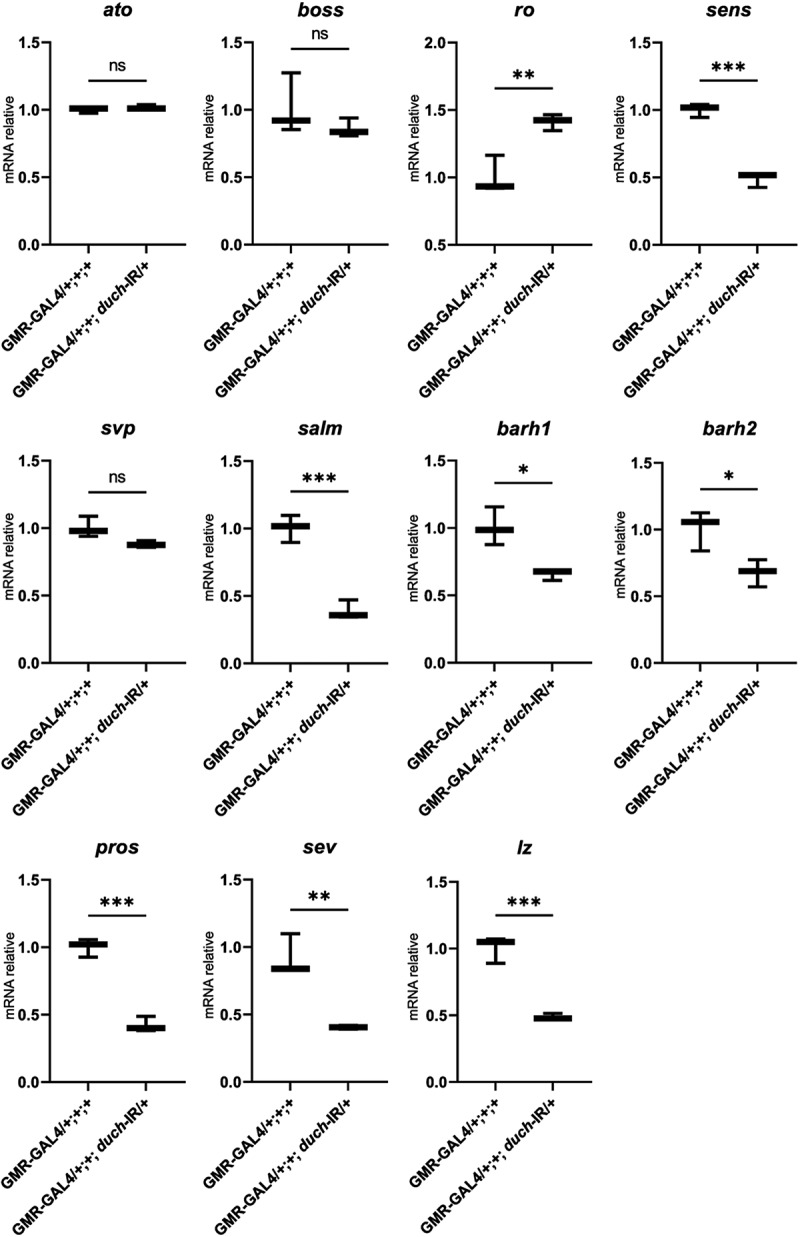
The expression of photoreceptor cell-related factors in knockdown d*uch* line compared to control line. *t*-test with *ato (p*=0.31), *boss* (*p*=0.32), ro (*p*=0.009), *sens* (*p*=0.0003), *svp* (*p*=0.06), *salm* (*p*=0.001), *barh1* (*p*=0.01), *barh2* (*p*=0.03), *pros* (*p*=0.0003), *sev* (*p*=0.004), *lz* (*p*=0.001).

The transcription factors BarH1/2 (*barh1/2*) and Prospero (*pros*), both essential for the normal development of R1 and R6 photoreceptor cells, were downregulated in the duch knockdown eye imaginal disc compared to controls. In relation to R7 photoreceptor differentiation, not only were barh1/2 and prospero reduced, but the expression of sevenless (sev) – a key regulator of R7 development [27,38], and *lozenge (lz)* was also downregulated in *duch* knockdown flies (Figure 5). In contrast, the expression of *atonal (ato)* and *bride of sevenless (boss)*, which are particularly important for R8 formation, remained unchanged under low dUCH conditions.

**Figure 6.**
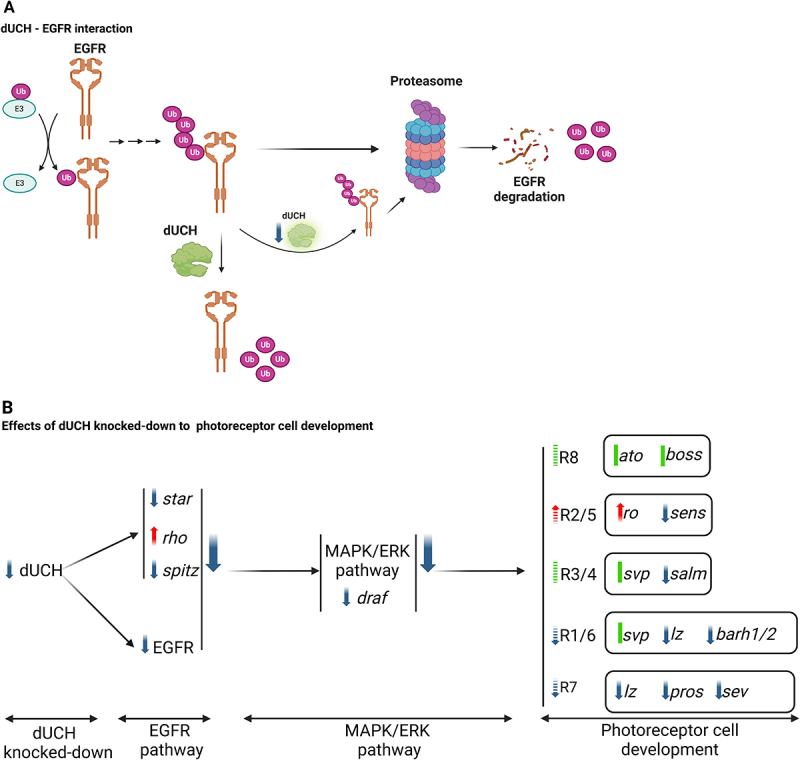
Diagram of dUCH and EGFR pathway involvement in *Drosophila* eye development. A. Interaction between dUCH and EGFR. B. Effects of dUCH knocked-down to photoreceptor cell differentiation.

Taken together, these results suggest that dUCH plays a significant role in photoreceptor cell differentiation (Figure 6). dUCH appears to regulate EGFR protein levels and is involved in the MAPK signalling pathway. Furthermore, it also modulates the expression of several key transcription factors that govern the differentiation of specific photoreceptor cell types (Figure 6).

## Discussion

UCH-L1 has been implicated in various conditions, including neurodegenerative diseases, diabetes, and cancer. Previous studies have shown that abnormal UCH-L1 function – resulting from mutations or altered protein expression levels – can cause multiple defects in living tissues. These findings also suggest that UCH-L1 influences several cellular processes, such as cell proliferation, cell cycle regulation, and cell death [6,59,60]

Our experimental data demonstrated that knockdown of *dUCH*—the *Drosophila melanogaster* homolog of human UCH-L1—disrupts the mitogen-activated protein kinase (MAPK) pathway, interferes with eye development, and leads to a rough eye phenotype.

When dUCH knockdown is driven by Rh1-Gal4, which is known to be specifically expressed in outer photoreceptors R1–R6, the result is a loss of eye pigmentation without any accompanying rough-eye phenotype. This outcome is consistent with the expression pattern of Rh1-Gal4: according to Charlière et al. (2022), Rh1-Gal4 is strictly confined to R1–R6 photoreceptors and does not drive expression in other eye cell types, such as pigment cells or interommatidial cells. Thus, dUCH knockdown via Rh1-Gal4 predominantly affects photoreceptor-specific processes – such as pigment synthesis or retention – without disrupting the structural integrity of ommatidia, hence no rough phenotype is observed. In contrast, when dUCH knockdown is performed using GMR-Gal4, a driver with broad expression across the entire eye including photoreceptors and pigment/interommatidial cells, the outcome is strikingly different. This is because dUCH is suppressed in all eye cell types, thus impacting processes critical for both pigmentation and morphological patterning.

The differential outcome loss of pigmentation only versus combined pigmentation loss and rough morphology can be attributed to the spatial specificity of the drivers. The observations support the idea that dUCH plays distinct roles in photoreceptor pigmentation versus global eye morphogenesis.

Given that UCH-L1 is an important component of the ubiquitin-proteasome system (UPS), and previous reports suggesting interactions between EGFR and the UPS [61–64]. Specifically, *d-Cbl*, an E3 ubiquitin ligase in *Drosophila*, has been shown to mediate EGFR internalization and degradation, playing significant roles in *Drosophila* eye development [65,66]. Besides, Bi et al. demonstrated a direct interaction between UCH-L1 and EGFR, showing that UCH-L1 binds to and stabilizes EGFR, preventing its degradation [67]. Consistent with this, our experimental results revealed that loss of dUCH function led to a reduction in EGFR protein levels without affecting its mRNA expression. Unexpectedly, we did not observe a rescue of the rough eye phenotype when conducted additional experiments using the UAS-EGFR line to overexpress EGFR in the background of dUCH loss-of-function. Despite the increased EGFR levels, dUCH knockdown flies still exhibited a rough eye (supplementary Fig. S2). One possible explanation is that EGFR was overexpressed at a level that was too high, which itself can cause a rough eye phenotype. As a result, in this experimental setup, we were unable to fine-tune EGFR expression to an appropriate level that could compensate for the reduction caused by dUCH knockdown.

The EGFR signalling pathway is well-known for its crucial role in photoreceptor cell differentiation [25,40,43,68,69]. Our data showed that *duch* knockdown severely affected the fly eye imaginal disc, including a reduction in *Draf*, a key MAPK pathway protein downstream of EGFR, important for R7 cell specialization. This prompted us to further investigate the role of dUCH loss in regulating EGFR components and photoreceptor differentiation. Interestingly, other EGFR pathway components, such as *rhomboid*, *star*, and the ligand *spitz*, were also affected by *duch* knockdown. Consistent with the reduced expression of *spitz* and EGFR, *draf* was downregulated as well. Several transcription factors regulating photoreceptor differentiation, including *senseless (sens)*, *spalt major (salm)*, *BarH1/2 (barh1/2)*, and *lozenge (lz)*, were downregulated under dUCH loss-of-function conditions. Since *senseless* is directly repressed by *rough (ro)*—a key factor for R2/R5 differentiation [41], the observed upregulation of *ro* in dUCH knockdown logically corresponds with the reduced *sens* expression.

*Spalt major (salm)* is critical for R3/4 development [41–44], while *BarH1/2* is essential for R1/6 differentiation [60,61]. Genes involved in R7 specialization—*barh1/2*, *prospero (pros)*, *lozenge (lz)*, and *sevenless (sev)*—were also downregulated, indicating that dUCH loss likely disrupts R7 development [27,37,38,70,71]. In contrast, genes crucial for R8 formation, such as *atonal (ato)* and *bride of sevenless (boss)*, remained unaffected despite reduced EGFR levels. This aligns with previous reports that EGFR signalling is not involved in R8 differentiation [43,57,58,68].

Interestingly, under dUCH knockdown conditions, although EGFR protein levels are reduced, we observe an increase in rhomboid1 (rho1) mRNA expression. This apparent paradox can be rationalized by considering feedback mechanisms within the EGFR signalling pathway and insights from spatial-temporal regulation during development. As outlined by Shilo (2005), rho1 transcription often prefigures sites of EGFR activation and plays a central role in launching localized waves of signalling within the eye disc or embryonic ectoderm. Under normal conditions, activated EGFR signalling exerts transcriptional feedback to suppress upstream components such as rho-1, in order to tightly control the spatial and temporal activation of the pathway [55]. However, when EGFR protein is destabilized due to impaired deubiquitination in the absence of dUCH, this feedback inhibition is likely lost. As a result, cells may transcriptionally upregulate rho-1 in a compensatory attempt to restore EGFR signalling. Additionally, previous studies have demonstrated that rho-1 expression is modulated not only by EGFR signalling itself but also by other regulatory inputs, including transcription factors like Pointed (Pnt), a downstream effector of the MAPK pathway. Impairment of MAPK signalling caused by reduced EGFR could further alter the regulation of rho-1, possibly leading to derepression and increased mRNA expression.

Importantly, the ubiquitin pathway plays a fundamental role in regulating protein stability, trafficking, and signalling during eye development. As a deubiquitinating enzyme, UCH-L1 removes ubiquitin moieties from target proteins, including mono-ubiquitin, thereby maintaining a sufficient pool of free mono-ubiquitin essential for normal cellular homoeostasis. In the context of eye development, mono-ubiquitin availability may be especially critical in regulating the turnover and localization of key receptors and transcription factors. It is plausible that reduced mono-ubiquitin levels caused by dUCH knockdown impair the proper trafficking of EGFR, shifting the balance towards lysosomal degradation rather than recycling, as mono-ubiquitination plays a key role in determining this fate [5,72–75].

Moreover, mono-ubiquitin dynamics are also known to influence chromatin remodelling and gene expression. For instance, mono-ubiquitination of histone H2A and H2B is associated with transcriptional regulation [76], suggesting that disrupted mono-ubiquitin pools in dUCH-deficient flies may lead to widespread transcriptional defects, including downregulation of genes involved in photoreceptor differentiation.

In support of this, the deubiquitinating enzyme Usp5 regulates Notch and RTK signalling during Drosophila eye development. Loss of Usp5 impairs photoreceptor development by downregulation RTK signalling, including EGFR [50]. UBPY mediated deubiquitination of EGFR promotes receptor degradation and affects MAPK signalling [74]. Loss of UCH-L1/dUCH activity likely results in a reduced availability of mono-ubiquitin, leading to dysregulation of ubiquitin-dependent processes. This could explain the observed decrease in EGFR protein levels, as mono-ubiquitination is known to modulate endocytic recycling versus degradation decisions.Moreover, altered mono-ubiquitin dynamics may also affect chromatin remodelling or transcriptional regulation of eye-specific genes, further compounding the developmental defects.

Taken together, the findings of this study highlight the critical role of the interaction between UCH-L1 and the EGFR signalling pathway in eye cell differentiation. Loss of dUCH function led to a reduction in EGFR protein levels, which in turn downregulated Draf in the MAPK pathway. Consequently, several key genes involved in photoreceptor cell differentiation were also affected by dUCH knockdown (Figure 6).

## Supplementary Material

Supplemental Material

## Data Availability

Data is available upon reasonable request.

## Submission declaration and verification

The authors declare that the work has not been published elsewhere.
