# Supplementary figures and images for: UCH-L1 regulates eye differentiation-related genes and modulates EGFR signalling in Drosophila melanogaster
Source: Fly (Austin). 2025 Nov 13;19(1):2580003. doi: 10.1080/19336934.2025.2580003 (PMC12622323; doi:10.1080/19336934.2025.2580003)

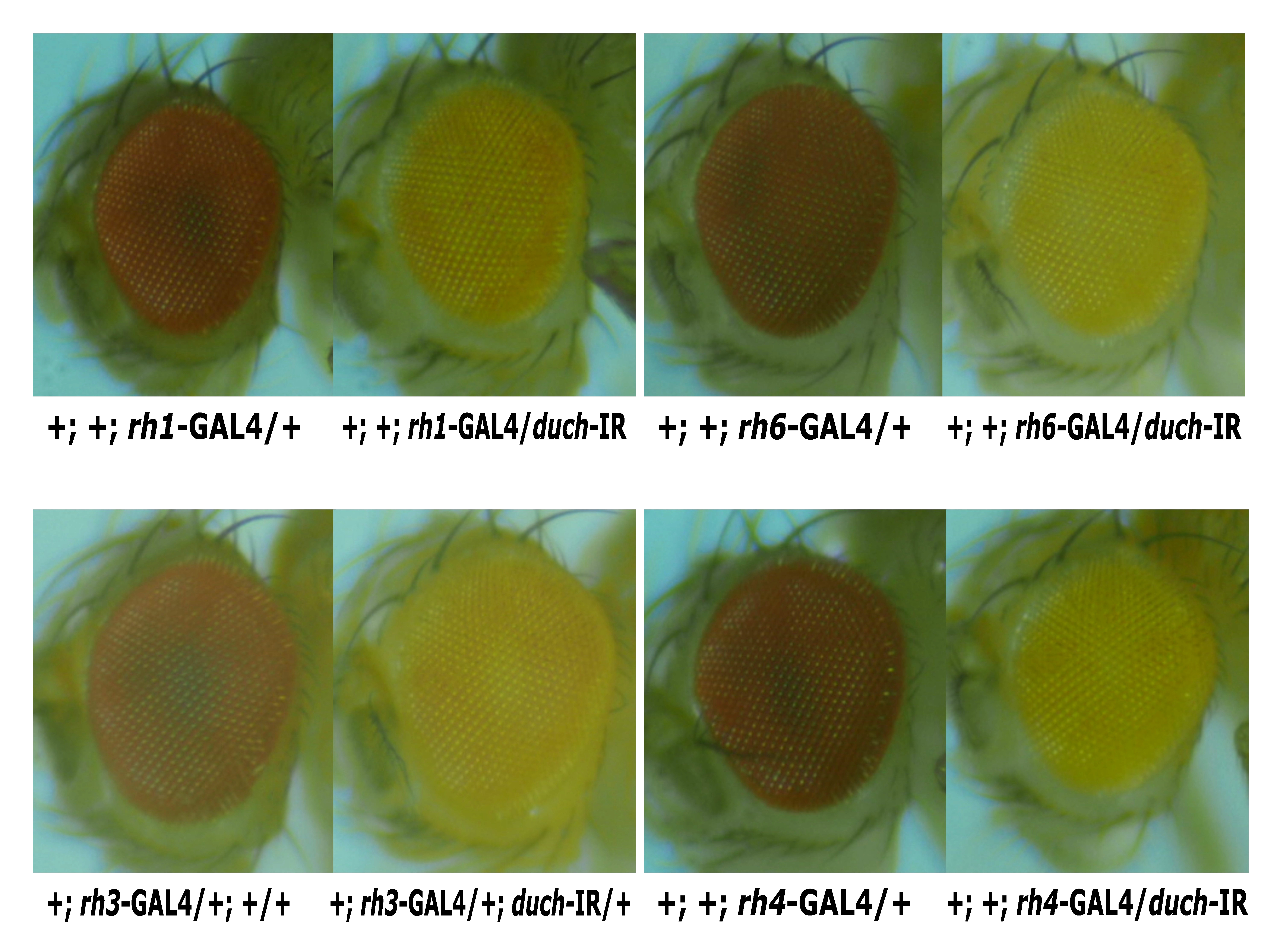

Supplement: Supplemental Material [file KFLY_A_2580003_SM7568.zip › Fig S1.jpg]

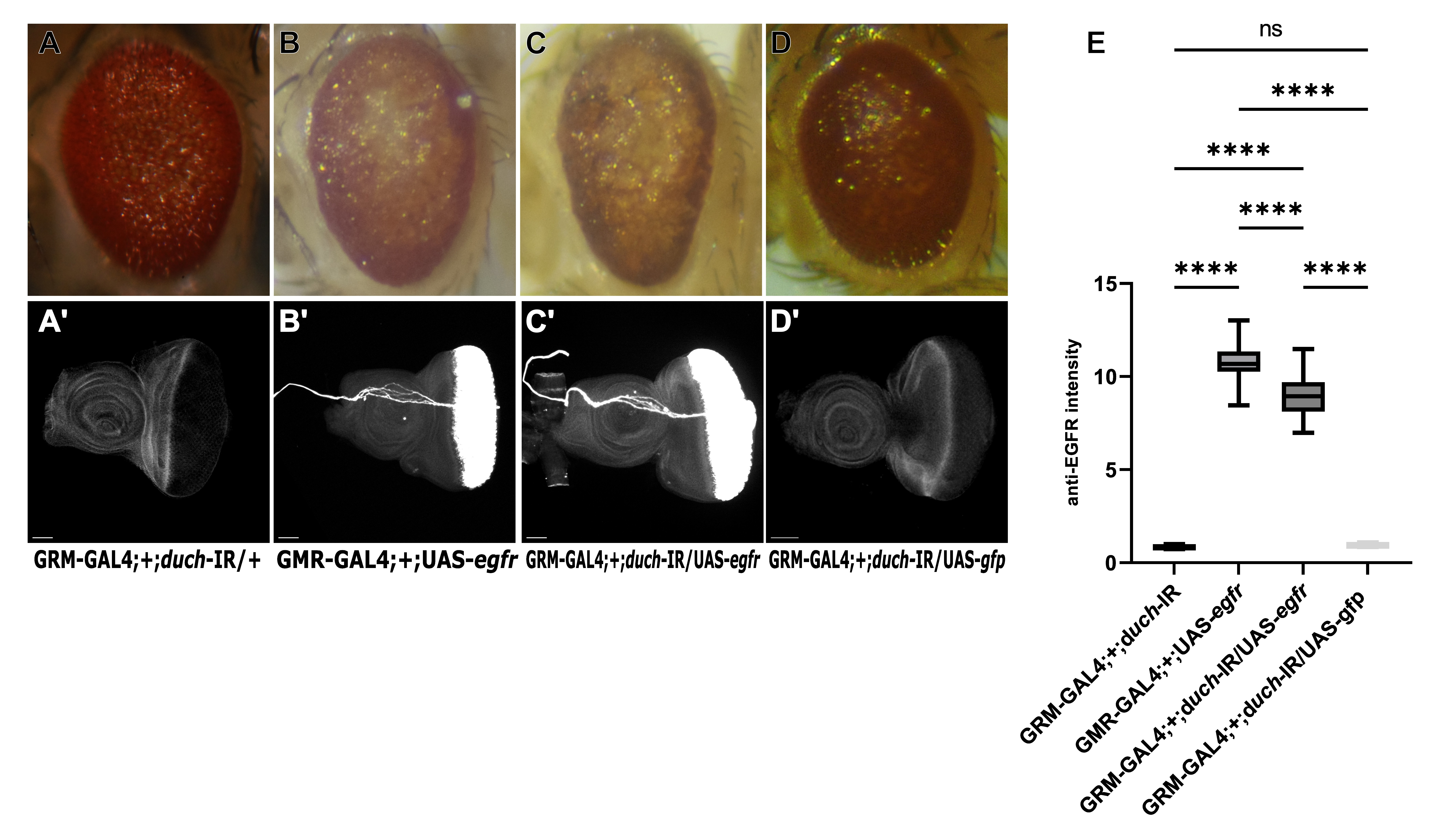

Supplement: Supplemental Material [file KFLY_A_2580003_SM7568.zip › Fig S2.jpg]
